# Effects of Astaxanthin on Ovarian Development of Largemouth Bass (*Micropterus salmoides*)

**DOI:** 10.1155/anu/2662809

**Published:** 2024-11-28

**Authors:** Mingwei Tao, Hangxian Zhou, Jie Wei, Qiyou Xu

**Affiliations:** ^1^College of Life Science, Huzhou University, Huzhou, China; ^2^Nation Local Joint Engineering Laboratory of Aquatic Animal Genetic Breeding and Nutrition, Zhejiang Provincial Key Laboratory of Aquatic Bioresource Conservation and Development Technology, Huzhou, China

**Keywords:** astaxanthin, largemouth bass, ovarian development, steroid

## Abstract

The purpose of the study was to investigate the effects of astaxanthin on ovarian development of largemouth bass (*Micropterus salmoides*) female. Five isonitrogenous and isolipidic feeds with varying levels of astaxanthin (0.8, 19, 41, 97, and 200 mg/kg) were grouped as AS0, AS20, AS40, AS100, and AS200, respectively. The results indicated that the gonadosomatic index (GSI) was significantly greater in the AS40 and AS100 than in AS0 and AS200 (*p* < 0.05). The AS40 and AS100 exhibited a dramatically lower hepatosomatic index (HSI) compared to the other groups (*p* < 0.05). The content of vitellogenin (VTG) was significantly increased in AS100 compared to the AS0, AS20, and AS200 (*p* < 0.05). Testosterone (T) levels were significantly lower in the AS200 compared to the other groups (*p* < 0.05). The AS40, AS100, and AS200 groups exhibited significantly greater follicle-stimulating hormone (FSH) levels than the AS0 and AS20 group (*p* < 0.05). The luteinizing hormone (LH) level was significantly higher in AS100 compared to the other groups (*p* < 0.05). The estradiol (E_2_) levels were significantly higher in AS40 compared to the AS0 and AS200 groups (*p* < 0.05). The total antioxidant capacity (T-AOC) was significantly higher in AS100 than the AS0 and AS20 groups (*p* < 0.05). The superoxide dismutase (SOD) activity was significantly higher in AS40 compared to the AS0 and AS200 groups (*p* < 0.05). The malondialdehyde (MDA) level was significantly decreased in AS40 than the other groups (*p* < 0.05). Transcriptomic analysis of ovarian tissue revealed that differentially expressed genes primarily involved in pathways such as “ovarian steroidogenesis,” “steroid hormone biosynthesis,” and “arachidonic acid metabolism.” The expression of genes involved in ovarian steroidogenesis and arachidonic acid metabolism, such as cytochrome P450 family 2 subfamily J member (*cyp2j*), insulin-like growth factor 1 (*igf1*), phospholipase A2 group (*pla2g*), FSH receptor (*fshr*), and acute regulatory protein (*star*), was significantly upregulated in the AS40 group (*p* < 0.05). In summary, appropriate amount of astaxanthin supplementation in the diet enhance gonadal development, antioxidant capacity, and sex hormone levels, promote the expression of genes related to gonadal development, and consequently, enhance reproductive performance of largemouth bass.

## 1. Introduction

For fish, the nutrient supply in the reproductive stage is important for the ovarian development, gamete quality, and larval growth of broodstock [1]. The ovaries can only develop into maturity if a certain level of nutritional substances is accumulated; otherwise, the oocytes will undergo atresia [2]. Carotenoids can improve egg quality and reduce embryonic mortality [3]. Astaxanthin, a red carotenoid pigment with strong antioxidant properties, must be obtained through diet by fish and crustaceans as they cannot synthesize it themselves [4]. In aquaculture, the focus on astaxanthin is mainly due to its ability to enhance body color, boost immune capacity, and improve antioxidant functions the of fish [5–8]. Recent studies have demonstrated that astaxanthin can enhance the reproductive performance of aquatic animals. The inclusion of 280 mg/kg astaxanthin in the feed significantly improves the gonadal maturation and hatching rate of black tiger shrimp (*Penaeus monodon*) [9]. The diet supplemented with 150 mg/kg of astaxanthin resulted in reduced oxidative stress in ovarian tissues, decreased cellular apoptosis, and enhanced oocyte development of female Nile tilapia (*Oreochromis niloticus*) [10]. However, the mechanism which astaxanthin influences reproduction remains unclear.

Largemouth bass (*Micropterus salmoides*), originally native to California, USA, and also known as the California bass, was introduced to the Guangdong, China, in the 1980s. With its short cultivation period, strong disease resistance, and broad temperature tolerance, it has gradually emerged as one of the key economic species in China's aquaculture industry over the years [11]. However, few studies have explored the impact of astaxanthin on broodstock reproductive performance, and currently, there is no research on its effect on largemouth bass ovarian development. Based on this, the study investigated the impacts and mechanisms of dietary astaxanthin supplementation on ovarian development of largemouth bass, aiming to provide data to support the formulation of compound diets for largemouth bass broodstock.

## 2. Materials and Methods

This research was conducted strictly according to the guidance of the care and use of laboratory animals in China. The experimental protocol was approved by Huzhou University's Committee on the Ethics of Animal Experiments (20220916).

### 2.1. Preparation of Experimental Feed

The astaxanthin (10%) was provided by Zhejiang NHU Company Ltd., with the remaining 90% consisting of chemically synthesized lignosulfonate, sugars, starch, and moisture.

In this study, a feed formula with isonitrogenous (47%) and lipidic (7%) content was designed based on the influence of astaxanthin on the ovarian development of Nile tilapia (*O. niloticus*) [10], goldfish (*Carassius auratus*) [12], and black tiger shrimp (*P. monodon*) [9], incorporating astaxanthin at levels of 0, 50, 100, 200, and 400 mg/kg. The actual contents measured were 0.8 (AS0), 19 (AS20), 41 (AS40), 97 (AS100), and 200 (AS200) mg/kg, respectively. The formulations and nutritional components of the experimental feeds are presented in Table 1. Fish meal, *α*-starch, soy protein concentrate, and soybean meal were ground, and passed through a 60-mesh screen, while vitamin premix, mineral premix, and calcium dihydrogen phosphate were passed through a 80-mesh screen. The components were mixed using a progressive augmentation technique. Astaxanthin was subsequently added, along with soybean oil, soy lecithin, and fish oil, and thoroughly mixed. The blend was formed into 4 mm diameter pellets using a twin screw extruder (F-26, South China University of Technology, Guangzhou, China) [13]. The pellets were dried at 40°C, and then kept at −20°C until use.

### 2.2. Feeding Management

This experiment was conducted in the circulating water culture system of the College of Life Sciences, Huzhou University. Before the start of the experiment, the largemouth bass were sterilized and disinfected, then placed in PE tanks (diameter × height = 1080 mm × 1200 mm) for temporary acclimatization (2 weeks). Then, 200 female largemouth bass with an initial average body weight of 438.58 ± 9.84 g were selected and randomly assigned five groups, with four tanks per group and 10 fish per tank, and a chip was injected into the muscle of each fish as a marker (make each fish as a parallel). Largemouth bass were hand-fed (satiation feeding) twice daily at 8:00 a.m. and 5:00 p.m. Feeding levels were adjusted every 2 weeks. Thirty minutes after feeding, the feeding behavior of the largemouth bass was observed, and any leftover diet in the aquaculture water system was collected. The experiment lasted for 35 days. Natural light and ventilation, and 1/3 water was changed daily. The water temperature was kept between 21 and 23°C, with dissolved oxygen ≥6 mg/L and ammonia nitrogen ≤0.3 mg/L.

### 2.3. Sample Collection

At the end of the experiment, two experimental fish from each tank, for a total of eight fish per group, which had developed to stage V, were randomly selected. The fish were placed on an ice tray, and blood was drawn from the caudal vein. The blood was placed into 1.5 mL centrifuge tubes and centrifuged at 3500 rpm for 10 min at the 4°C after keeping in refrigerator at 4°C for 4 h. The supernatant was aspirated as serum and stored at −80°C for subsequent biochemistry examination. Under sterile conditions, the ovarian and liver were excised and separated, and their weights were accurately measured and recorded. Liver and ovarian tissues were promptly stored in liquid nitrogen for short-term preservation before being moved to a freezer at −80°C for genetic and biochemical examination. Another part of the ovary and liver was embedded in 10 mL centrifuge tubes, fixed with formaldehyde reagent, and stored at room temperature for histological observation. Additionally, 0.1 g of eggs (from three replicates) was taken from each fish for egg diameter (ED) calculation.

### 2.4. Indicators and Measuring Methods

The drying method was used to measure the moisture content at 105°C (GB/T 6435-2006/ISO 6496:1999). The Dumas nitrogen analyzer was used to calculate the crude protein content (GB/T 6432-94). The Soxhlet extractor was used to determine crude fat content (GB/T 6443-2006/ISO 6492:1999). Ash content was measured using a 550°C muffle furnace (GB/T 6438-2007/ISO 5984:2002). Astaxanthin content in the feed was measured by Shandong Bayong Biotechnology Co., Ltd. The samples were extracted with a mixed solution of dichloromethane and dimethyl sulfoxide, and then cholesterol esterase was added. After the enzymatic hydrolysis, anhydrous sodium sulfate and petroleum ether were added for centrifugation, dried with nitrogen, dissolved in acetone, and passed through a 0.22 μm (Nylon) organic filter. After separation by C30 reversed-phase liquid chromatography column, it was determined by liquid chromatography.

The total antioxidant capacity (T-AOC), superoxide dismutase (SOD), and malondialdehyde (MDA) levels in the liver were measured using kits from Nanjing Jiancheng Bioengineering Institute. The contents of ovarian vitellogenin (VTG), serum estradiol (E_2_), follicle stimulating hormone (FSH), luteinizing hormone (LH), and testosterone (T) were determined by the kit produced by Jiangsu Meimian Industrial Co., Ltd.

### 2.5. HE Sections of Liver and Ovarian Tissue

Liver and gonadal tissues were fixed at air temperature for 24 h in formaldehyde solution and then processed by Hangzhou Hulk Biotechnology Co., Ltd. Prepared histological sections were observed under an optical microscope to visually examine vacuolization, sinusoidal congestion, and nuclear displacement in liver tissue sections [14, 15]. Follicular cells and atresia follicles at different stages in the ovary were counted under a fourfold magnifying glass [16].

### 2.6. Ovarian Transcriptome Analysis

Ovarian transcriptome analysis of AS0 group and AS40 (four samples in each group) was performed by Shanghai Meiji Biotechnology Co., Ltd. for RNA extraction, quality control, library construction, and sequencing. RSEM software was utilized to quantify transcript and gene expression levels. After obtaining the read counts for genes, differential expression was analyzed using the DESeq2 software, with the default criteria for significantly differentially expressed genes being: FDR < 0.05 and |log2FC| ≥ 1. The Python scipy package was used to statistically analyze the enrichment of genes that were differentially expressed in KEGG and GO pathways.

### 2.7. Quantitative RT-PCR (qRT-PCR) Analysis

In order to verify the results of RNA-Seq sequencing analysis, six differentially expressed genes were selected for qRT-PCR analysis. According to the Unigene sequence obtained by sequencing, primers were designed using primer premier 6 software (Table 2), and *β*-actin gene was used as an internal reference. The UltraSYBR Mixture kit (Kangwei Century) was used to carry out real-time qPCR. The comparative CT method (2^–*ΔΔct*^) was employed to calculate the relative levels of gene expression [17].

### 2.8. Statistical Analysis

The experimental data were analyzed using SPSS 25.0 software for one-way ANOVA. Group comparisons were performed using the Duncan method, with differences considered significant at *p* < 0.05. The results were presented as mean ± standard error (mean ± SE).

The calculation formula is as follows:

  $$\begin{array}{c} \text{Gonadosomatic index }\left(\text{GSI},\text{ \% }\right)=\text{Gonad wet weight}/\text{body wet weight}\times 100. \end{array}$$

  $$\begin{array}{c} \text{Hepatosomatic index }\left(\text{HSI},\text{ \% }\right)=\text{Hepatopancreas wet weight}/\text{body wet weight}\times 100. \end{array}$$

  $$\begin{array}{c} \text{Absolute fecundity }\left(\text{AF},\text{gr}\right)=\text{Number of eggs in the sample}/\text{sample wet weight}\times \text{gonad wet weight}. \end{array}$$

## 3. Results

### 3.1. Effects of Astaxanthin on Ovarian Morphology and Reproductive Performance in Largemouth Bass

**Figure 1:**
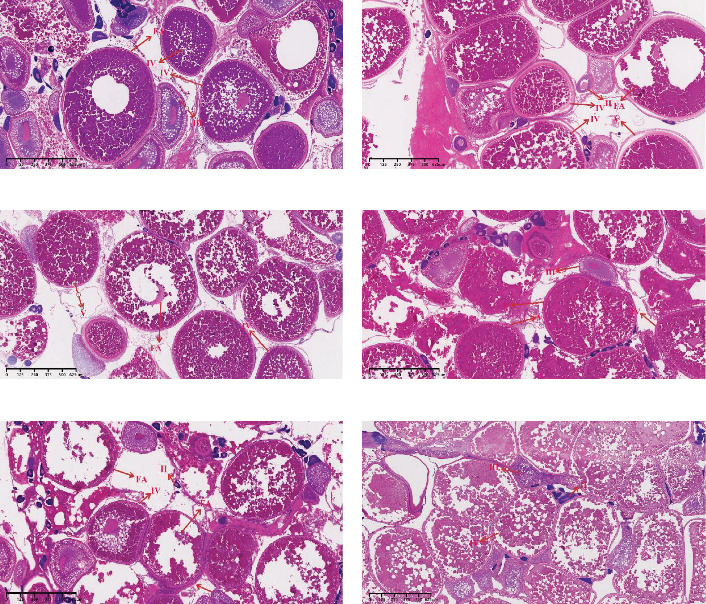
Oocyte development in largemouth bass fed with diets containing different levels of astaxanthin for 35 days. Representative images of HE stained gonad tissue from female fish fed with diets containing astaxanthin (magnification ×4, scale bar: 625 μm). (a)–(e) corresponded to AS0, AS20, AS40, AS100, and AS200 groups, respectively. (f) corresponds to the initial gonad. II, III, IV, and V represent oocytes at stages II, III, IV, and V, respectively. AS0, 0.8 mg/kg astaxanthin; AS20, 19 mg/kg astaxanthin; AS40, 41 mg/kg astaxanthin; AS100, 97 mg/kg astaxanthin; AS200, 200 mg/kg astaxanthin; FA, follicular atresia; HE, hematoxylin/eosin.

As shown in Figure 1 and Table 3, histological observations of gonadal sections from different groups of largemouth bass were conducted. The results of hematoxylin/eosin (HE) staining showed that in the initial gonads of largemouth bass, the proportions of oocytes at different developmental stages and atretic follicles were as follows: stage II oocytes accounted for 34%, stage III oocytes for 20%, stage IV oocytes for 34%, stage V oocytes for 0%, and atretic follicles for 12%. On the end of experiment, the proportions of oocytes at different developmental stages were: In the AS0 group, stage II oocytes 28%, stage III 28%, stage IV 28%, stage V 5%, and atretic follicles 11%. In the AS20 group, stage II oocytes accounted for 27%, stage III oocytes for 13%, stage IV oocytes for 33%, stage V oocytes for 20%, and atretic follicles for 6%. In the AS40 group, stage II oocytes accounted for 18%, stage III oocytes for 9%, stage IV oocytes for 18%, stage V oocytes for 36%, and atretic follicles for 18%. In the AS100 group, stage II oocytes accounted for 41%, stage III oocytes for 9%, stage IV oocytes for 16%, stage V oocytes for 25%, and atretic follicles for 9%. In the AS200 group, stage II oocytes accounted for 31%, stage III oocytes for 12%, stage IV oocytes for 25%, stage V oocytes for 6%, and atretic follicles for 25%.

**Figure 2:**
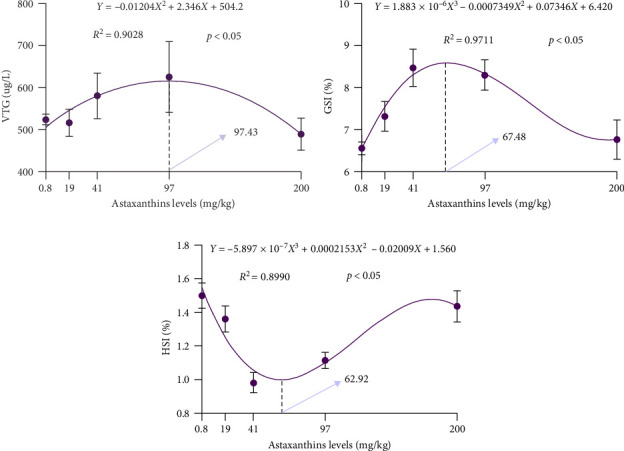
Effects of astaxanthin on ovarian development and reproductive performance of largemouth bass. (a)–(c) correspond to VTG, GSI, and HSI, respectively. GSI, gonadosomatic index; HSI, hepatosomatic index; VTG, vitellogenin.

According to Table 4, it is observed that the five groups did not exhibit any significant differences in absolute fecundity (AF) and ED (*p* > 0.05). With the increase of astaxanthin levels in the feed, the hepatosomatic index (HSI) exhibited a trend of initially decreasing and then increasing, reaching its lowest point in the AS40 group and was significant differences compared to the AS0, AS20, and AS200 groups (*p* < 0.05). The gonadosomatic index (GSI) initially increased and then decreased, peaking in the AS40 group, which showed significant differences compared to the AS0 and AS200 groups (*p* < 0.05). With astaxanthin in the feed increased, ovarian VTG content peaked in the AS100 group and was significantly different with the AS0, AS20, and AS200 groups (*p* < 0.05). It showed in curve fitting (Figure 2) that the maximum VTG content in the ovaries was attained at 97.43 mg/kg astaxanthin, the peak GSI was reached at 67.48 mg/kg, and the lowest HSI occurred at 62.92 mg/kg.

### 3.2. Effects of Astaxanthin on Serum Hormones of Largemouth Bass

**Figure 3:**
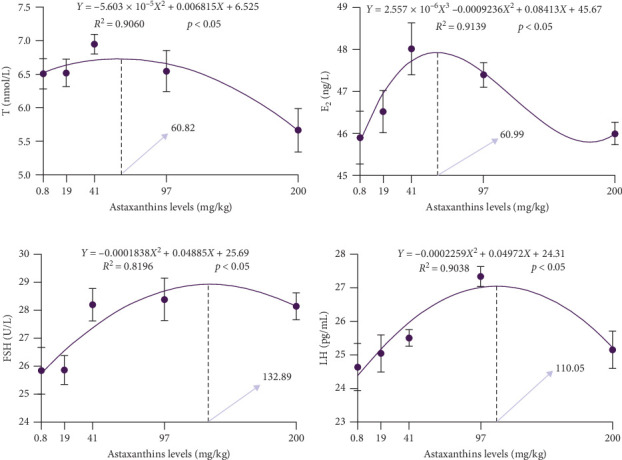
Effects of astaxanthin on serum hormones of largemouth bass. (a)–(d) correspond to T, E_2_, FSH, and LH, respectively. E_2_, estradiol; FSH, follicle stimulating hormone; LH, luteinizing hormone; T, testosterone.

As indicated by Table 5, significant changes in serum E_2_, T, FSH, and LH was observed with varying levels of astaxanthin in the diet (*p* < 0.05). The serum E_2_ levels in the AS40 group were significantly higher than those in the AS0 and AS200 groups (*p* < 0.05). Serum T levels were significantly lower in the AS200 group than the other groups (*p* < 0.05), and there was no significant difference among the remaining groups. Serum levels of FSH were significantly higher in the AS40, AS100, and AS200 groups than in the other two groups (*p* < 0.05). Serum LH levels were significantly higher in the AS100 group than in the other groups (*p* < 0.05). It revealed in curve fitting (Figure 3) that the highest serum T level was achieved at the 60.82 mg/kg astaxanthin, the highest serum E_2_ level at 60.99 mg/kg, the highest FSH level at 132.89 mg/kg, and the highest LH level at 110.05 mg/kg.

### 3.3. Effects of Astaxanthin on Liver Damage and Antioxidation in Largemouth Bass

**Figure 4:**
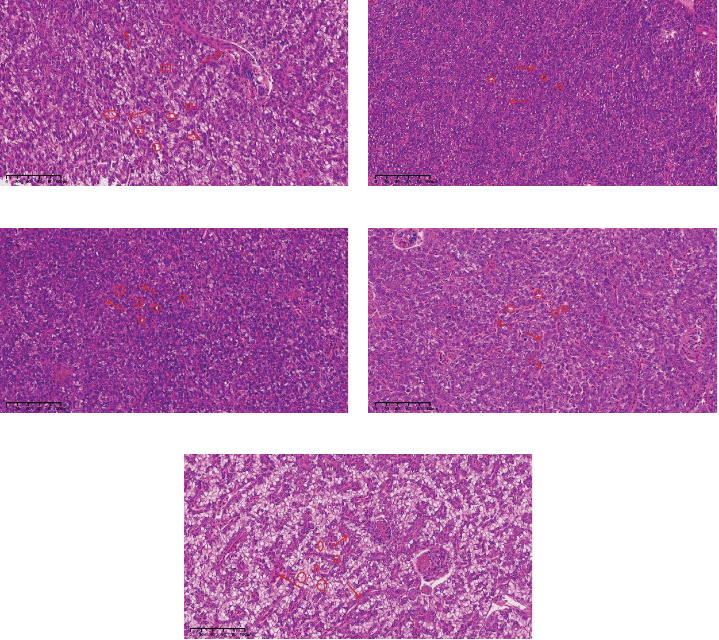
Hepatic histology of largemouth bass fed with diets containing different levels of astaxanthin for 35 days. Representative images of HE, stained liver tissue from female fish fed with diets containing astaxanthin (magnification ×20, scale bar: 100 μm). (a)–(e) corresponded to AS0, AS20, AS40, AS100, AS200 feed groups, respectively. Arrow: sinusoidal conestion; ellipse: cytoplasmic vocuolization; square: nucleus. AS0, 0.8 mg/kg astaxanthin; AS20, 19 mg/kg astaxanthin; AS40, 41 mg/kg astaxanthin; AS100, 97 mg/kg astaxanthin; AS200, 200 mg/kg astaxanthin; HE, hematoxylin/eosin.

Histological examinations of liver were presented in Figure 4. HE staining revealed that, compared to the AS0 group, the AS20, AS40, and AS100 groups showed a reduced vacuolation rate and vacuolar area in the liver. Additionally, hepatocyte morphology normalized, and the boundaries between hepatocytes became progressively clearer. However, when the level of astaxanthin increased to 200 mg/kg, improvements in the morphological structure of the liver was not observed, there was even a displacement or disappearance of the nucleus, and an increased frequency of sinusoid congestion was observed.

As shown in Table 6, the liver SOD activity initially increased, then decreased with higher astaxanthin levels, peaking in the AS40 group and significantly differing from the AS0 and AS200 groups (*p* < 0.05). Similarly, the liver T-AOC capacity increased and then decreased with rising levels of astaxanthin in the feed, reaching its highest in the AS100 group, which showed significant differences from the AS0 and AS20 groups (*p* < 0.05). The liver MDA content showed a decrease and then an increase, reaching its lowest level in the AS40 group, which was significantly different from the other groups (*p* < 0.05).

### 3.4. Transcriptome Analysis of the Effect of Astaxanthin on Ovarian

**Figure 5:**
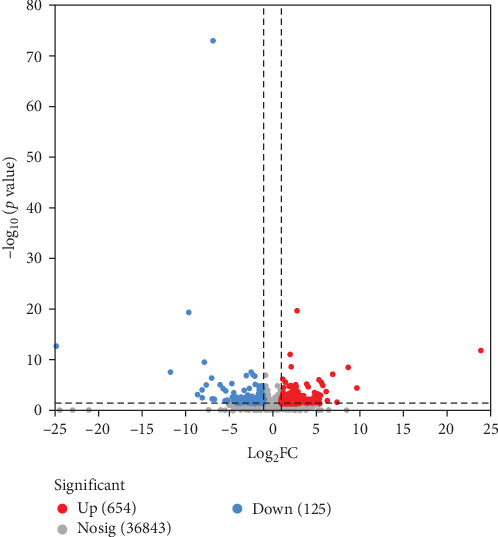
The number of differentially expressed genes in ovarian tissue of largemouth bass between the AS0 group and the AS40 group. AS0, 0.8 mg/kg astaxanthin; AS40, 41 mg/kg astaxanthin.

**Figure 6:**
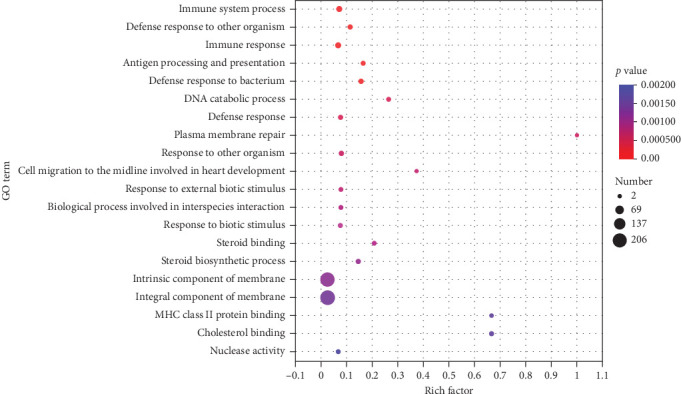
GO classification of differentially expressed genes in the gonad tissues between the AS0 group and the AS40 group. AS0, 0.8 mg/kg astaxanthin; AS40, 41 mg/kg astaxanthin.

**Figure 7:**
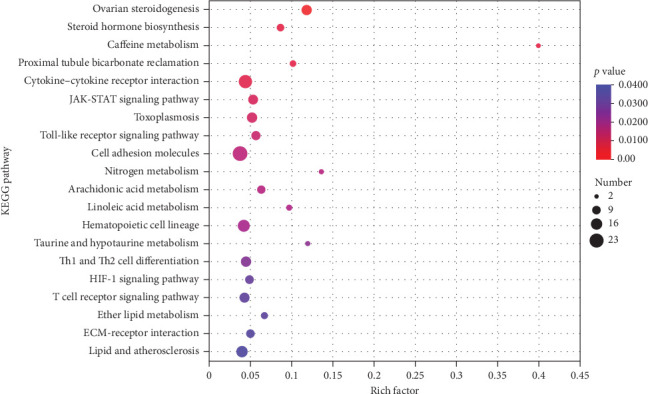
KEGG enrichment subclasses and signaling pathways of differentially expressed genes in the gonad tissues between the AS0 group and the AS40 group. AS0, 0.8 mg/kg astaxanthin; AS40, 41 mg/kg astaxanthin.

Transcriptomic analysis was indicated by Table 7, the quality control data for the eight samples showed sequencing error rates between 0.0125% and 0.0128%, Q20 ranged from 98.16% to 98.39%, Q30 from 94.66% to 95.26%, and GC contents between 47.92% and 49.18%. These results confirm that the sequencing data of the eight samples meet the standards for transcriptomic analysis. The statistical results of DEGs in the ovaries of largemouth bass were displayed in Figure 5, where 654 genes were upregulated and 125 genes were downregulated between the AS0 group and the AS40 group. To elucidate the physiological regulatory mechanisms of ovarian development in largemouth bass, a GO enrichment analysis of DEGs was performed (Figure 6), which detects significant associations with processes such as “immune system process,” “defense response to other organism,” and “immune response” (*p* < 0.05). Furthermore, for analyzing the biological functions of ovarian development, a Kyoto Encyclopedia of KEGG pathway enrichment analysis of DEGs was conducted (Figure 7). The results suggest that pathways such as “ovarian steroidogenesis,” “steroid hormone biosynthesis,” and “arachidonic acid metabolism” (*p* < 0.05) influence the development of the ovaries in largemouth bass.

### 3.5. DEGs Were Verified by Fluorescence qPCR

**Figure 8:**
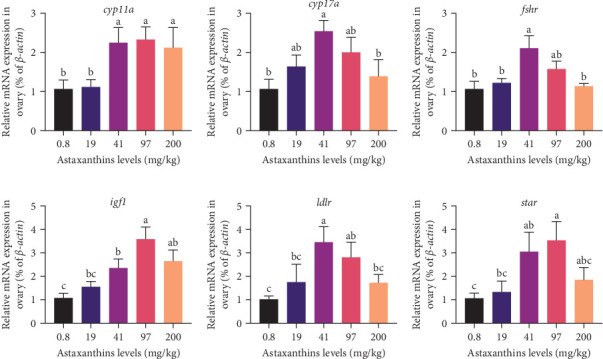
(a–f) The transcription levels of genes related to ovarian steroid pathway of largemouth bass fed with feed containing astaxanthin for 35 days. All data were expressed as mean ± SE (*n* = 4). Mean values within the same row with different lower superscripts showed significant differences (*p* < 0.05). *cyp11a*, cytochrome P450 family 11 subfamily A member; *cyp17a*, cytochrome P450 family 17 subfamily A member; *fshr*, follicle stimulating hormone receptor; *igf1*, insulin-like growth factor 1; *ldlr*, low-density lipoprotein receptor; SE, standard error; *star*, acute regulatory protein.

To confirm the reliability of the transcriptomic data, six DEGs from the significantly enriched pathways were validated using qPCR analysis. The results were presented in Figure 8, compared with the AS0 group, FSH receptor (*fshr*), cytochrome P450 family 17 subfamily A member (*cyp17a*), acute regulatory protein (*star*), cytochrome P450 Family 11 Subfamily A Member (*cyp11a*), low-density lipoprotein receptor (*ldlr*), and insulin-like growth factor 1 (*igf1*) were significantly elevated in the AS40 group (*p* < 0.05). This consistency demonstrated that the transcriptomic sequencing analysis results were accurate and reliable.

## 4. Discussions

The main nutritional elements involved in the development of broodstock ovaries include proteins, lipids, vitamins, minerals, and trace elements. The fundamental purpose of researching is to explore how varying nutritional ratios affect ovarian development. It has been found by Chong et al. [18] that protein content between 50% and 60% in the feed significantly enhances the relative fecundity and fry production rate of Green swordtail (*Xiphophorus hellerii*). Research by Du et al. [19] has discovered that a lipid content of 16.31% in the feed significantly increases the serum levels of T and E_2_ in Chinese sturgeon (*Acipenser sinensis*). Furthermore, it has been identified by Furuita et al. [20] that adding 337 × 10^3^ IU/100 g of vitamin A to the feed improves the hatching rate of rainbow trout (*Oncorhynchus mykiss*). Therefore, supplementing a suitable amount of nutrient elements into the feed can enhance the reproductive performance of the broodstock.

The reproductive performance indicators of broodstock include VTG content, GSI, HSI, and serum hormone levels. VTG, a high-density lipoprotein complex, serves as a nutritional source for embryos and early-stage larvae [21, 22], and it provides a direct indication of gonadal development. Our experiment indicated that the highest levels of VTG and GSI occurred when the actual astaxanthin addition was between 40 and 100 mg/kg, and these levels were inversely related to the HSI. The primary reason was: when the oocytes were in the middle to late stages of yolk formation (stages IV and V), the synthesis of exogenous VTG occurs, which is produced in the liver, transported to the ovaries, and binds to VTG receptors on the oocytes, thus, providing nutrition to the developing embryos [20, 23].

The content of steroid hormones in broodstock is mainly determined by the speed of production in tissues such as gonads and the speed of metabolism in blood [24]. It has been found in studies [25] that steroid hormones regulate the growth, development, and maturation of gonads in vertebrates via the hypothalamus–pituitary–gonadal (HPG) axis, with the main fish steroid hormones including E_2_, T, progesterone (P), and cortisol (C). In this study, the AS40 group exhibited significantly greater levels of E_2_, this is consistent with findings by Abdel-Ghani et al. [26], Xu et al. [27], and Qiang et al. [10] in studies on cows, Tongue Sole (*Cynoglossus semilaevis*), and tilapia (*Oreochromis mossambicus*). Conversely, T levels was notably lower in the AS200 group, which may be attributed to excessive astaxanthin causing oocyte developmental arrest, thereby affecting ovarian development. Gonadotropins were important glycoprotein hormones (GPHs) in fish, which can promote the growth, development, and maturation of oocytes [28, 29]. There were two kinds of gonadotropins in fish, LH and FSH, both classified as glycoproteins [30]. FSH and LH reach the ovaries through the bloodstream and bind to receptors distributed on the ovaries to exert different physiological functions. Serum levels of FSH and LH were highest in the AS100 group, and HE staining analysis shows abundant stage V oocytes in the ovaries of both the AS40 and AS100 astaxanthin groups. Existing research has indicated that supplementing with astaxanthin can enhance serum hormone levels (FSH and LH) and improve the T-AOC capacity of ovaries in aged hens [31], aligning with the findings of this study. These findings suggest that adding suitable amounts of astaxanthin can elevate serum levels of FSH and LH, thereby, accelerating yolk production, oocyte development, and maturation.

The liver is not only the central organ of physiological metabolism in the body but also processes and transports synthesized nutrients to the ovaries [32]. The body's antioxidant system removes excess free radicals and active oxygen to prevent oxidative damage, and the liver's antioxidant defense is especially sensitive to environmental and nutritional changes [33]. MDA, resulting from lipid peroxidation due to free radicals, indicates the severity of cellular damage [34]. T-AOC measures the body's total antioxidant capacity, capable of scavenging free radicals and active oxygen to maintain normal physiological functions [35]. The activity of SOD can indirectly reflect the capability to eliminate oxygen free radicals [36]. Therefore, the content of T-AOC, MDA, and SOD activity can be utilized to evaluate the organism's antioxidant capacity. It has been found that astaxanthin possesses strong antioxidative properties, which can be attributed to the presence of conjugated double bonds, hydroxyl groups, and ketone groups in its molecular structure. These groups can neutralize endogenous free radicals and convert them into more stable compounds, thereby, ending the free radical chain reactions within the organism [37–39]. Research by Cheng and Wu [40] indicated that the actual addition of 200–800 mg/kg of astaxanthin significantly enhances SOD capacity and reduces MDA content in the hepatopancreas of red swamp crayfish (*Procambarus clarkii*). Similarly, Jiang et al. [41] discovered that the hepatopancreatic T-AOC capacity of the Chinese mitten crab (*Eriocheir sinensis*) was significantly improved, and MDA content was significantly decreased by the actual addition of 60–90 mg/kg of astaxanthin. Similar to these findings, the results of this study indicate that supplementing with an actual additional amount of 40–100 mg/kg of astaxanthin significantly enhances SOD activity and T-AOC capacity and reduces MDA content in the liver of largemouth bass. This implies that adding astaxanthin to the feed improves the liver's antioxidative capacity, thereby, protecting the synthesis of lipids (phospholipids), proteins, amino acids, and VTG, which may be crucial for improving reproductive performance.

In the field of developmental biology focusing on fish, transcriptomic sequencing technology is extensively utilized in areas such as animal embryology and artificial reproduction [42, 43]. KEGG functional enrichment analysis had indicated that many differentially expressed genes were concentrated in pathways such as “ovarian steroidogenesis,” “steroid hormone biosynthesis,” and the “arachidonic acid metabolism pathway.” Arachidonic acid, a component of cell membrane phospholipids, is released when the cell is activated. Through the activity of cyclooxygenases (*cox*), lipoxygenases (*lox*), and the cytochrome P450 enzyme system (*cyp*), it is converted into various compounds with high physiological activity. These compounds play significant physiological roles in the development of gonads [44–46]. It has been found in this study that in the AS40 group, astaxanthin can upregulate genes such as *pla2g* and *cyp2j*. The primary function of *pla2g* is to release arachidonic acid from cell membrane phospholipids, while *cyp2j* further oxidizes free arachidonic acid to produce prostaglandins [44]. In fish, prostaglandins play a role in oocyte maturation, development, and the synthesis of sex hormones [47, 48]. Research on crabs has demonstrated that injections of prostaglandins significantly increase the GSI and oocyte diameter [49]. Studies on Giant River Prawn (*Macrobrachium rosenbergii*) have also found that prostaglandins can shorten the ovarian cycle, increase oocyte proliferation, and enhance VTG content [50]. The above findings demonstrated that the metabolism of arachidonic acid was activated after largemouth bass ingested astaxanthin, and the synthesis of prostaglandins was promoted by related factors, thus, accelerating ovarian development in the fish.

The transformation of cholesterol into estrogen within follicles is a complex process primarily regulated by the ovarian steroid metabolism pathway, involving genes such as *fshr*, *cyp11a*, *cyp17a*, *cyp19a1*, *star*, and *igf1* [51]. This study found that the expression levels of ovarian steroid-related genes in the AS40 group increased compared to the AS0 group and corresponded with the trends in E_2_ changes. Similar findings was reported by Aiceles, Gombar, and da Fonte Ramos [52] where consistent changes in serum E_2_ and steroidogenesis-related gene expression was found in mice. Cholesterol, vital for steroid survival, is transformed into pregnenolone in the ovaries by the action of the *cyp11a*. Pregnenolone, a precursor to many sex hormones (E_2_, P, and T), is transformed into these sex hormones through a series of enzymes (*3β-hsd*, *cyp17a*, and *cyp19*). These sex hormones (E_2_) stimulate the formation of VTG in the liver, which is essential for oocyte development; thus, the upregulation of *cyp17a* and *cyp11a* can enhance the synthesis of sex hormones [53, 54]. *igf1* is a critical regulatory factor for oocyte mitosis, differentiation, apoptosis, embryonic development, and growth regulation [55, 56]. Synthesized sex hormones exert endocrine feedback on the release of gonadotropins, affecting the function of the hypothalamus and pituitary gland, thereby, regulating the further production of sex hormones [57, 58]. As a cofactor of gonadotropin, *igf1* will stimulate oocytes to produce sex hormones and promote oocyte proliferation together with FSH. Research by Maestro et al. [59] found that *igf1* plays a role in regulating ovarian steroidogenesis in Coho Salmon (*Oncorhynchus keta*) prior to ovulation. Studies by Srivastava and Van Der Kraak [60] revealed that *igf1* and gonadotropins were involved in VTG and DNA synthesis in follicles of goldfish (*C. auratus*). *Star* regulates the entry of cholesterol into mitochondria, a critical step in steroid biosynthesis [61]. Research on mammals shows that gonadotropins and *igf1* can regulate steroidogenesis by stimulating the expression of *star* [62]. *fshr*, in female fish, is primarily associated with VTG and oocyte maturation. In studies conducted on zebrafish (*Danio rerio*) and European sea bass (*Dicentrarchus labrax*), it was observed that the expression level of *fshr* gradually increases during the late stages of VTG and oocyte maturation [63, 64]. In rainbow trout (*O. mykiss*), *fshr* expression mainly participates in the early VTG process [65]. Based on the analysis above, it is inferred that the intake of astaxanthin by largemouth bass activates the ovarian steroid pathway. Consequently, it is hypothesized that the intake of astaxanthin by largemouth bass increases the levels of sex hormones and gonadotropins, thereby, accelerating the development of largemouth bass ovaries.

## 5. Conclusions

Astaxanthin supplementation can enhance the gonadal development, serum hormone levels, and antioxidant capacity of largemouth bass. Moreover, it can upregulate genes associated with the “ovarian steroidogenesis” and “arachidonic acid metabolism.” Based on the results of this research, and using GSI, VTG, HSI, and E_2_ as indicators, 60.99–97.43 mg/kg of astaxanthin is recommended in the broodstock formulated diet. This provide new insights in the mechanisms by which astaxanthin influences the ovarian development of largemouth bass.

## Figures and Tables

**Table 1:** Ingredients and proximate compositions of the experimental diets (%, dry weight).

Item (%)	AS0	AS20	AS40	AS100	AS200
Fish meal	40	40	40	40	40
Soy protein concentrate	22	22	22	22	22
Soybean meal	12	12	12	12	12
Fish oil	2.5	2.5	2.5	2.5	2.5
Soybean oil	2.5	2.5	2.5	2.5	2.5
Soy lecithin	2	2	2	2	2
*α*-Starch	11	11	11	11	11
Vitamin mix^a^	0.5	0.5	0.5	0.5	0.5
Mineral mix^b^	0.5	0.5	0.5	0.5	0.5
Choline chloride	0.5	0.5	0.5	0.5	0.5
Carboxymethyl cellulose	2	2	2	2	2
Microcrystalline cellulose	2.5	2.45	2.4	2.3	2.1
Ca(H_2_PO_4_)_2_	2	2	2	2	2
Astaxanthin (10%)	0	0.05	0.1	0.2	0.4
Total	100	100	100	100	100
Proximate composition					
Moisture (%)	7.07	6.79	6.87	6.41	6.29
Crude protein (%)	47.08	46.82	47.68	47.90	48.33
Crude lipid (%)	7.81	7.32	6.36	7.22	6.40
Ash (%)	10.92	10.63	10.43	10.72	10.61
Astaxanthin (mg/kg)	0.8	19	41	97	200

Abbreviations: AS0, 0.8 mg/kg astaxanthin; AS20, 19 mg/kg astaxanthin; AS40, 41 mg/kg astaxanthin; AS100, 97 mg/kg astaxanthin; AS200, 200 mg/kg astaxanthin.

^a^Vitamin premix provided the following per kg of diets: Vitamin A: 16,000 IU, Vitamin C: 150 mg, Vitamin D_3_: 2000 IU, Vitamin E: 360 mg, Vitamin K_3_: 10 mg, Vitamin B_1_: 16 mg, Vitamin B_2_: 45 mg, Vitamin B_6_: 20 mg, Vitamin B_12_: 0.4 mg, Calcium pantothenate: 70 mg, nicotinamide: 80 mg, folic acid: 5 mg, biotin: 1 mg, inositol: 320 mg, zeolite meal: 3886.6 mg.

^b^Mineral premix provided the following per kg of diets: FeSO_4_·7H_2_O: 124.13 mg, CuSO_4_·5H_2_O: 9.77 mg, MnSO_4_·H_2_O: 26.15 mg, ZnSO_4_·7H_2_O: 154.53 mg, Na_2_SeO_3:_ 0.44 mg, Ca(IO_3_)_2_: 2.31 mg, CoCl_2_·6H_2_O: 1.6 mg, MgSO_4_·7H_2_O: 1224.49 mg, zeolite meal: 3456.59 mg.

**Table 2:** Nucleotide sequences of the primers used to assay gene expressions by real-time PCR.

Gene	Primer sequence (5′−3′)	Gene accession number
*fshr*	GGTACTACAACCACGCCATAGACTG TAACAGCCGCACACGCAGAAG	XM_038736037.1
*Igf1*	TCCGCTGTGGTACTGACCTCCT TCTGCTGGTGGTGGTAATTCTCCTT	XM_038738328.1
*cyp17a*	AATGTCATCTGTTCGCTCTGCTTCA TGAGGAGGTGGTCGTCACTGAG	XM_038731415.1
*star*	ATGTCTTATGTGAAGCAAGGTGAGGAT AGGTAGGTCCATTCTCCGCTCTG	XM_038702459.1
*cyp11a*	TTGCTGGATGAAGTTGGTGAGGATT CGTAACTGCCTGTAGATGTTATGGATG	XM_038706496.1
*ldlr*	TCAAGATGGATCGGACGAGGTCAA GCACGGCAGGAGCAGTTATAGC	XM_038694104.1
*β-actin*	GCCCCACCTGAGCGTAAATA AGCTGAAGTTGTCGGGTGTT	XM_038736037.1

Abbreviations: *cyp11a*, cytochrome P450 family 11 subfamily A member; *cyp17a*, cytochrome P450 family 17 subfamily A member; *fshr*, follicle stimulating hormone receptor; *igf1*, insulin-like growth factor 1; *ldlr*, low-density lipoprotein receptor; *star*, acute regulatory protein.

**Table 3:** Ovarian development stage of largemouth bass.

Item	Ⅱ (%)	Ⅲ (%)	Ⅳ (%)	Ⅴ (%)	FA (%)
IG	34	20	34	0	12
AS0	28	28	28	5	11
AS20	27	13	33	20	6
AS40	18	9	18	36	18
AS100	41	9	16	25	9
AS200	31	12	25	6	25

*Note:* II, III, IV, and V represent oocytes at stages II, III, IV, and V, respectively.

Abbreviations: AS0, 0.8 mg/kg astaxanthin; AS20, 19 mg/kg astaxanthin; AS40, 41 mg/kg astaxanthin; AS100, 97 mg/kg astaxanthin; AS200, 200 mg/kg astaxanthin; FA, follicular atresia; IG, initial gonadal state.

**Table 4:** Effects of astaxanthin on ovarian development and reproductive performance of largemouth bass.

Item	Astaxanthin level (mg/kg)					PSE	*p* values	Linear	Quadratic	Cubic
	AS0	AS20	AS40	AS100	AS200					
VTG (ug/l)	525.258^b,c^	517.229^b,c^	581.788^a,b^	626.390^a^	490.101^c^	15.090	0.011	0.566	0.003	0.007
AF (gr /10^4^)	3.099	3.024	2.584	3.178	3.077	0.082	0.149	0.539	0.777	0.181
GSI (%)	6.571^b^	7.335^a,b^	8.490^a^	8.319^a^	6.779^b^	0.233	0.007	0.758	0.002	0.003
ED (mm)	1.236	1.243	1.222	1.209	1.216	0.0059	0.357	0.125	0.153	0.280
HSI (%)	1.500^a^	1.360^a^	0.979^b^	1.112^b^	1.435^a^	0.539	0.001	0.808	0.002	0.001

*Note:* All data were expressed as mean ± SE (*n* = 4). Mean values within the same row with different lower superscripts showed significant differences (*p* < 0.05).

Abbreviations: AF, absolute fecundity; AS0, 0.8 mg/kg astaxanthin; AS20, 19 mg/kg astaxanthin; AS40, 41 mg/kg astaxanthin; AS100, 97 mg/kg astaxanthin; AS200, 200 mg/kg astaxanthin; ED, egg diameter; GSI, gonadosomatic index; HSI, hepatosomatic index; SE, standard error; VTG, vitellogenin.

**Table 5:** Effects of astaxanthin on serum hormones of largemouth bass.

Item	Astaxanthin level (mg/kg)					PSE	*p* values	Linear	Quadratic	Cubic
	AS0	AS20	AS40	AS100	AS200					
E_2_ (ng/L)	45.905^b^	46.540^a,b^	48.042^a^	47.419^a,b^	46.001^b^	0.273	0.033	0.734	0.016	0.029
T (nmol/L)	6.504^a^	6.521^a^	6.946^a^	6.544^a^	5.665^b^	0.138	0.032	0.010	0.007	0.018
FSH (U/L)	25.840^b^	25.874^b^	28.204^a^	28.389^a^	28.149^a^	0.373	0.020	0.027	0.009	0.024
LH (pg/ml)	24.646^b^	25.047^b^	25.511^b^	27.336^a^	25.155^b^	0.294	0.015	0.425	0.004	0.005

*Note:* All data were expressed as mean ± SE (*n* = 4). Mean values within the same row with different lower superscripts showed significant differences (*p* < 0.05).

Abbreviations: AS0, 0.8 mg/kg astaxanthin; AS20, 19 mg/kg astaxanthin; AS40, 41 mg/kg astaxanthin; AS100, 97 mg/kg astaxanthin; AS200, 200 mg/kg astaxanthin; E_2_, estradiol; FSH, follicle stimulating hormone; LH, luteinizing hormone; SE, standard error; T, testosterone.

**Table 6:** Effect of astaxanthin on liver antioxidant of largemouth bass.

Item	Astaxanthin level (mg/kg)					PSE	*p* values	Linear	Quadratic	Cubic
	AS0	AS20	AS40	AS100	AS200					
MDA (nmol/mgprot)	9.978^a^	9.213^a^	7.575^b^	8.911^a^	9.540^a^	0.251	0.012	0.753	0.098	0.012
T-AOC (U/mgprot)	0.247^b^	0.255^b^	0.273^ab^	0.333^a^	0.293^a,b^	0.010	0.048	0.067	0.011	0.019
SOD (U/mgprot)	74.741^b^	81.383^a,b^	88.571^a^	86.512^a^	76.859^b^	1.691	0.019	0.758	0.008	0.008

*Note:* All data were expressed as mean ± SE (*n* = 4). Mean values within the same row with different lower superscripts showed significant differences (*p* < 0.05).

Abbreviations: AS0, 0.8 mg/kg astaxanthin; AS20, 19 mg/kg astaxanthin; AS40, 41 mg/kg astaxanthin; AS100, 97 mg/kg astaxanthin; AS200, 200 mg/kg astaxanthin; MDA, malondialdehyde; SE, standard error; SOD, superoxide dismutase; T-AOC, total antioxidant capacity.

**Table 7:** Quality assessment of the sequencing data of ovary samples in different treatments.

Sample	Raw reads	Raw bases	Clean reads	Clean bases	Error rate (%)	Q20 (%)	Q30 (%)	GC content (%)
AS0-1	52,961,882	7,997,244,182	52,422,550	7,829,816,371	0.0125	98.39	95.26	47.93
AS0-2	55,912,142	8,442,733,442	55,334,492	8,270,231,766	0.0126	98.27	94.97	48.95
AS0-3	59,443,608	8,975,984,808	58,812,388	8,773,203,480	0.0126	98.3	95.04	49.02
AS0-4	48,404,312	7,309,051,112	47,834,264	7,146,885,450	0.0128	98.16	94.66	49.18
AS40-1	53,879,906	8,135,865,806	53,363,378	7,977,682,984	0.0125	98.37	95.2	48.35
AS40-2	54,588,698	8,242,893,398	54,017,842	8,053,801,283	0.0126	98.28	94.99	49.27
AS40-3	50,088,792	7,563,407,592	49,572,498	7,409,773,760	0.0126	98.33	95.11	47.92
AS40-4	48,787,042	7,366,843,342	48,261,160	7,214,238,587	0.0126	98.32	95.06	48.27

Abbreviations: AS0, 0.8 mg/kg astaxanthin; AS40, 41 mg/kg astaxanthin.

## Data Availability

The data that support the findings of this study are available on request from the corresponding author. The data are not publicly available due to privacy or ethical restrictions.
